# Enhanced Resolution Analysis for Water Molecules in MCM-41 and SBA-15 in Low-Field T_2_ Relaxometric Spectra

**DOI:** 10.3390/molecules26082133

**Published:** 2021-04-08

**Authors:** Grzegorz Stoch, Artur T. Krzyżak

**Affiliations:** Department of Fossil Fuels, AGH University of Science and Technology, A. Mickiewicza Av., 30-059 Cracow, Poland; akrzyzak@agh.edu.pl

**Keywords:** low field NMR, Inverse Laplace Transform, L-Curve regularization, confined liquid, relaxometry, drying process

## Abstract

Mesoporous silica materials are the subjects for relaxometric NMR studies in which we obtain information on the properties of molecules in confined geometries. The signal analysis in such investigations is generally carried out with the help of the Inverse Laplace Transform (ILT), which is accompanied by a regularization procedure. The appropriate selection of the regularization method may positively affect the resolution of the spectrum and the essence of the final conclusions. In this work, we examined the MCM-41 and SBA-15 model systems in various saturation states, using L-Curve regularization for relaxation spectra based on our own version of the fast fast ILT implementation. In a single relaxometric spectrum, the water contributions from the internal volume in the pores and between the silica particles were identified, which allowed us to trace the dynamics of the corresponding drying trends during the removal of water from the sample as a function of total water saturation.

## 1. Introduction

Water molecules trapped in silica mesoporous materials behave differently than free water. This property can be used to study model systems with a developed surface, and the results might be extended to real systems. As shown by Grünberg et al. [1] with the help of ^1^H MAS NMR spectra, we are able to identify different water contributions in such materials due to different chemical environments for the surface water and the water from the pore’s interior space. What makes this identification feasible is standard Fourier transform (FT) methodology that splits the overall signal into groups of spins rotating with different Larmor frequencies, embodied in the frequency-domain spectrum.

A similar goal was achieved in our previous work [2] by means of a different tool and methodology where a series of a low-field ^1^H NMR measurements and time-domain analysis led to conclusions consistent with frequency domain analysis made earlier by Grünberg et al. [1]. Contributions were obtained using a combination of ILT together with the sample’s weight monitoring through a series of measurements at different water saturation. However, due to the line broadening inherent for ILT regularization, we have not been able to break down a single spectrum into the desired components, contrary to what is common in frequency domain methodology. Although NMR relaxometry is an established tool for the characterization of porous materials and its results are confirmed by independent measurements (e.g., using the gas adsorption and micro porosimetry methods [3,4]), its resolution is still a matter of progress.

In this article, we check whether the improved resolution of our transform will affect the ability to separate contributions and the ability to track their evolution in individual spectra for MCM-41 and SBA-15 nanoparticle systems. The resolution improvement was achieved through the implementation of the ILT algorithm from scratch and a revised regularization methodology. Before using it, the usefulness of the algorithm was assessed by comparing it with the one used so far in previous works. In this article, we use the term “ILT” as a convenient label to refer to the exponential nature of a signal, but the mathematically correct formulation for this is: the Fredholm problem of the first kind with an exponential kernel [5].

### 1.1. Importance of a Low Field, Bulk and Surface Signal from within a Pore

The FID (Free Induction Decay) signal is described by the relaxation time given [6,7] by

(1) $$\frac{1}{T_{2}^{*}}=\frac{1}{T_{2}^{B}}+\frac{1}{T_{2}^{S}}+\frac{1}{T_{2}^{P}}$$

where $T_{2}^{B}$ characterizes *free water*, $T_{2}^{S}$ is the surface relaxation term and $T_{2}^{P}$ is the influence of inhomogeneity of magnetic field on the signal that is of microscopic internal origin and might be additionally caused by external (macroscopic) field gradient, so that $\frac{1}{T_{2}^{P}}=\frac{1}{T_{2}^{act}}+\frac{1}{T_{2}^{D}}$ (where: $T_{2}^{act}$ is the actual refocusable term, and $T_{2}^{D}$ is the component describing diffusion caused by external field gradient *G*).

Application of the CPMG sequence [8] removes refocusable component $T_{2}^{act}$, which converts $T_{2}^{*}$ to $T_{2}$ by reducing $T_{2}^{P}$ in Equation (1) to the diffusion term $T_{2}^{D}$, which depends on the echo time *t_E_* [6] through the expression

(2) $$\frac{1}{T_{2}^{D}}=D\frac{{\left(\gamma Gt_{E}\right)}^{2}}{12}$$

This term can be minimized using short echo time $t_{E}$, which, together with the small B_0_ field magnitude [9], makes the last term in Equation (1) usually neglected, and we recorded the decay of the spin echo envelope, given by the effective expression in Equation (3).

(3) $$\frac{1}{T_{2}}=\frac{1}{T_{2}^{B}}+\frac{1}{T_{2}^{S}}$$

Brownstein and Tarr showed that the surface term in Equation (3) is given by the expression where geometrical details are reduced to a simple relationship between the pore’s surface *S* and its volume *V*:

(4) $$\frac{1}{T_{2}^{S}}=\rho \frac{S}{V}$$

which for the cylindrical shape of the pore of radius *r* takes the form:

(5) $$\frac{1}{T_{2}^{S}}=\rho \frac{2}{r}$$

They considered relaxing spins within a single pore interior, experiencing a diffusion effect [10], where spin-to-surface diffusion time is significantly shorter than the spin relaxation time $T_{2}^{S}$. Within this time, all molecules interact with the surface [1,11] and their fast exchange with the interior volume makes the entire magnetization for the pore uniform (so-called fast diffusion regime). This work concerns the results obtained in the fast exchange mode, and the expectation of separating the contributions $T_{2}^{B}$ and $T_{2}^{S}$ for materials with such small pores does not seem justified. Rather, we would expect to see coarser differences like those between the inside of the pores and their closest and other neighborhoods.

For cylindrical pores, Equation (5) is the basic way to differentiate the pore size based on the T_2_ spectrum and thus identify the individual water contributions. Small-sized pores correspond to shorter relaxation times, and larger pores to proportionally longer T_2_, therefore, in favorable but rare circumstances, the spectrum is a set of separate lines on the logarithmic T_2_ scale.

### 1.2. Our Mesoporous System and Experimental

**Figure 1:**
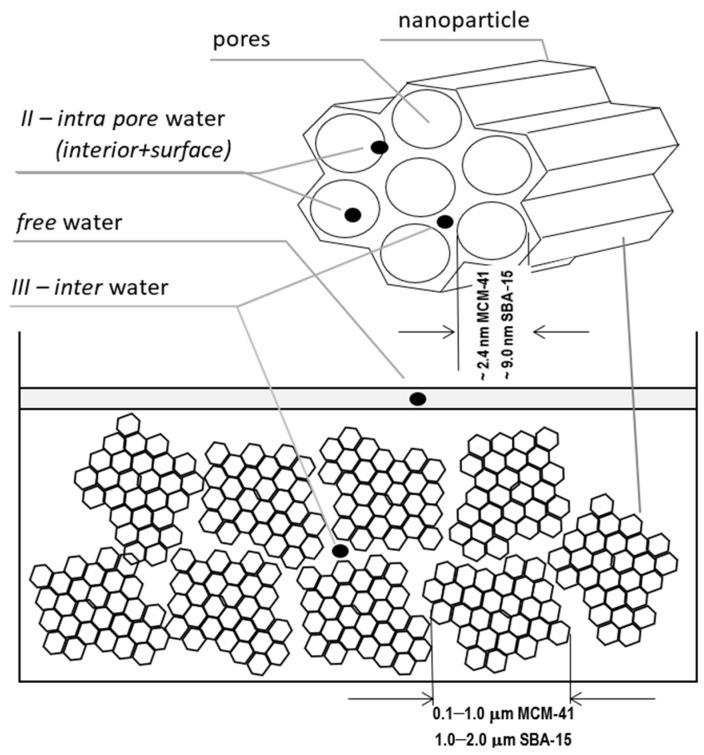
The considered locations for water placement: free water, water inside the pores (as the bulk and surface water), in-between the pores and nanoparticles.

The measured system was in the form of a powder of nanoparticles filled with demineralized water, which was dried in subsequent steps. The initial saturation was close to 100% and a small water film was left on the surface itself as a marker. The spaces we considered fundamental for water placement are shown in Figure 1. First, this is the water in pores we expect to identify in our spectra toward shorter relaxation times as unresolved according to internal intra pore water and surface water. Clusters of nanoparticles form a kind of dense gel in which a sort of external pore is formed between the clusters of nanoparticles, giving rise to a somewhat longer T_2_ together with water particles between the pores (III—inter-pore water). Finally, all of the free water including that left intentionally on the sample’s surface was characterized with the longest T_2_ time ~2 s.

The material for sample preparation was provided by Sigma Aldrich, had a structure of hexagonally arranged cylindrical pores, diameters ranging from 2.1 to 2.7 nm, average particle size of 0.1–1.0 μm for MCM-41 and 7.0 to 10.0 nm diameter, and 1–2 μm average particle size for SBA-15.

In the first step, the full saturation of the sample was obtained by adding 6.97 g of water to 0.99 g of the weighed dry powder. After completing the measurements for full saturation, the sample was dried at 80 °C in subsequent steps, each lasting 30–60 min. At each step, the sample was weighed (Radwag analytical balance, ±1 mg) and relaxation measurements repeated. Mass measurements enabled the determination of sample saturation states in the subsequent steps of the experiment based on mass balance.

Prior to hydration, the porous properties of silica were characterized by adsorption of N_2_, from where the pores’ surface area *S_BET_* = 1001 m^2^/g and their total volume $V_{tot}^{0.99}$ = 0.981 cm^3^/g per mass unit were obtained. At the end of the experiment, the characterization of the dried sample was repeated. A few percent decrease in the area of pores and volume was noted, which can be attributed to the slight hydrolysis effect [12]. However, unchanged diagram of the respective isotherm suggests [2] that the inner structure of the sample was retained in the filling and then in the successive drying processes. The nitrogen sorption isotherms at −196 °C were obtained by gas volumetry using a Micrometrics ASAP2020 analyzer in the relative pressure range of 10^−4^ to 0.99. The ^1^H NMR relaxation signal measurements were performed on a low-field 0.05 T Magritek Rock Core Analyzer with a 29 mm probe at 30 °C using the CPMG *t_E_* = 60 μs sequence with pulse length of 10 µs and 50,000 echoes.

Spectra were obtained from the collected signals using the ILT transform. The practical difference between the ILT and the more widely used Fourier transform (FFT) is the numerical instability inherent in the nature of the former as opposed to the latter [13]. Regularization is a necessary modification of the initial system of ILT equations so that this system does not become numerically ill-conditioned due to the presence of perturbations of various origins, first of all, experimental noise [14]. Regularization requires finding the so-called lambda parameter, which specifies the degree of regularization using separate criteria and is independent of the original ILT problem. These criteria (regularization algorithms) are subject to continuous improvement and the value of the lambda parameter affects the resolution of the ILT spectrum.

**Figure 2:**
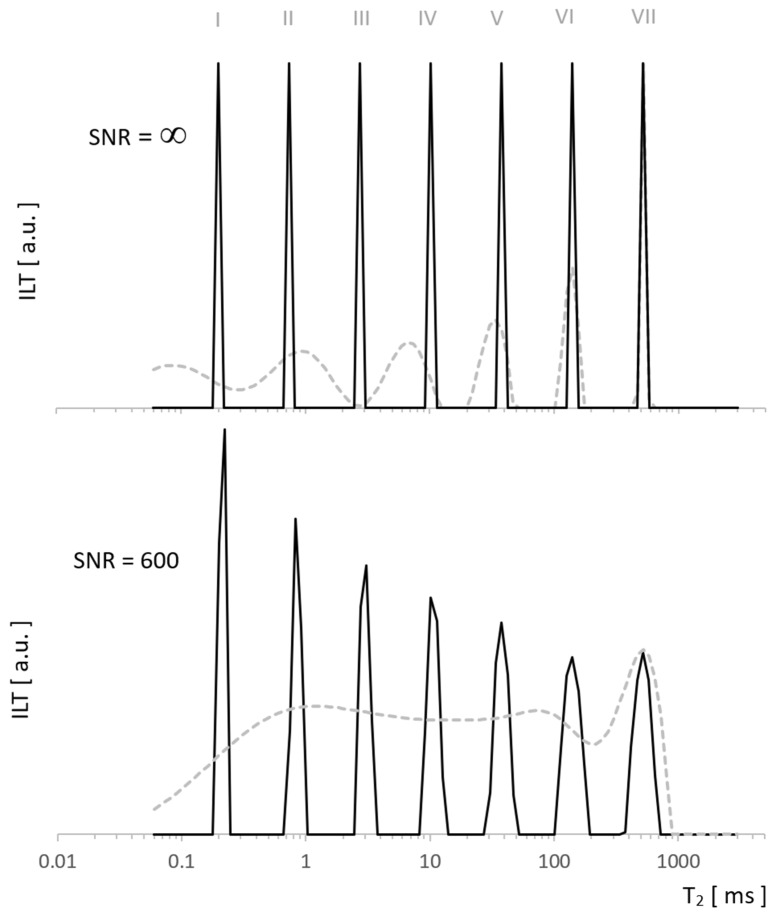
Spectra of the test signal with T_2_ distribution as seven Kronecker deltas. Obtained using ILT with L-Curve regularization (solid line) vs. other regularization method (dashed line) for noiseless data (upper figure) and for SNR = 600 (lower figure). Significant differences in the resolution resulted from the use of different regularization algorithms.

Our data were analyzed using the TNT-NN algorithm [15], which, being much faster than the classic Lawson–Hanson version [16], has a positive effect on the possibilities and reliability of computationally expensive regularization. We used the L-Curve method [17], which is less conservative than that of the RCA Toolbox Package used so far [18] and at the same time safer due to the risk of under-regularization compared to Generalized Cross Validation (GCV) [19,20]. Both L-Curve and *GCV* methods are well-established in a wide range of applications, in particular, L-Curve has been used successfully in tomography [21], the reconstruction of paintings [22], geoscience [23], and many other disciplines. For the test, we generated a pattern with which ILT fundamentally does not work well (sharp edges) to see the possibilities of the algorithm on especially demanding tasks. A comparison with the method we used so far [18] shows enhanced resolution for the new approach in Figure 2, both for data without noise and for SNR = 600. The former presents the exact reconstruction of the seven Kronecker delta patterns by the new algorithm, while the latter shows typical distortions for it, but still much smaller than in our standard approach.

## 2. Results and Discussion

**Figure 3:**
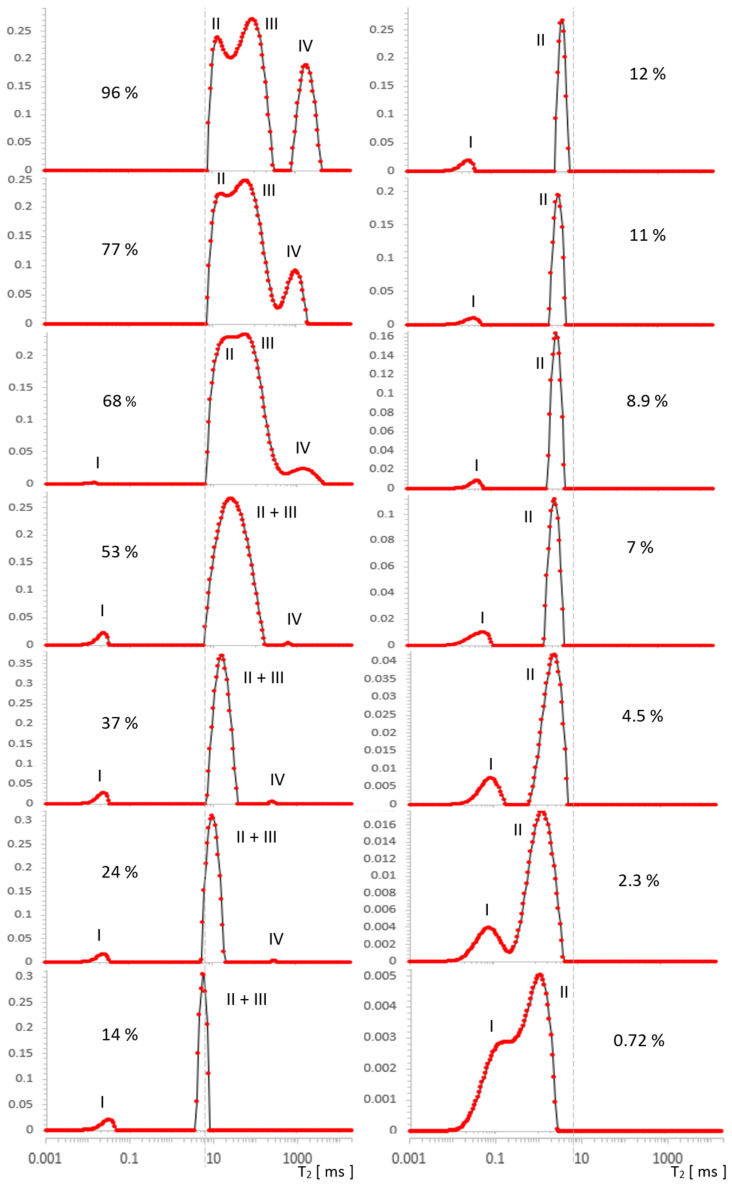
Evolution of T_2_ spectrum for MCM-41, obtained in [a.u.] using ILT transform, along a series of water saturations. I—OH groups, II—intra pore water, III—inter pore water, IV—free water. The vertical axes represent the values of the ILT transform in [a.u.] units.

**Figure 4:**
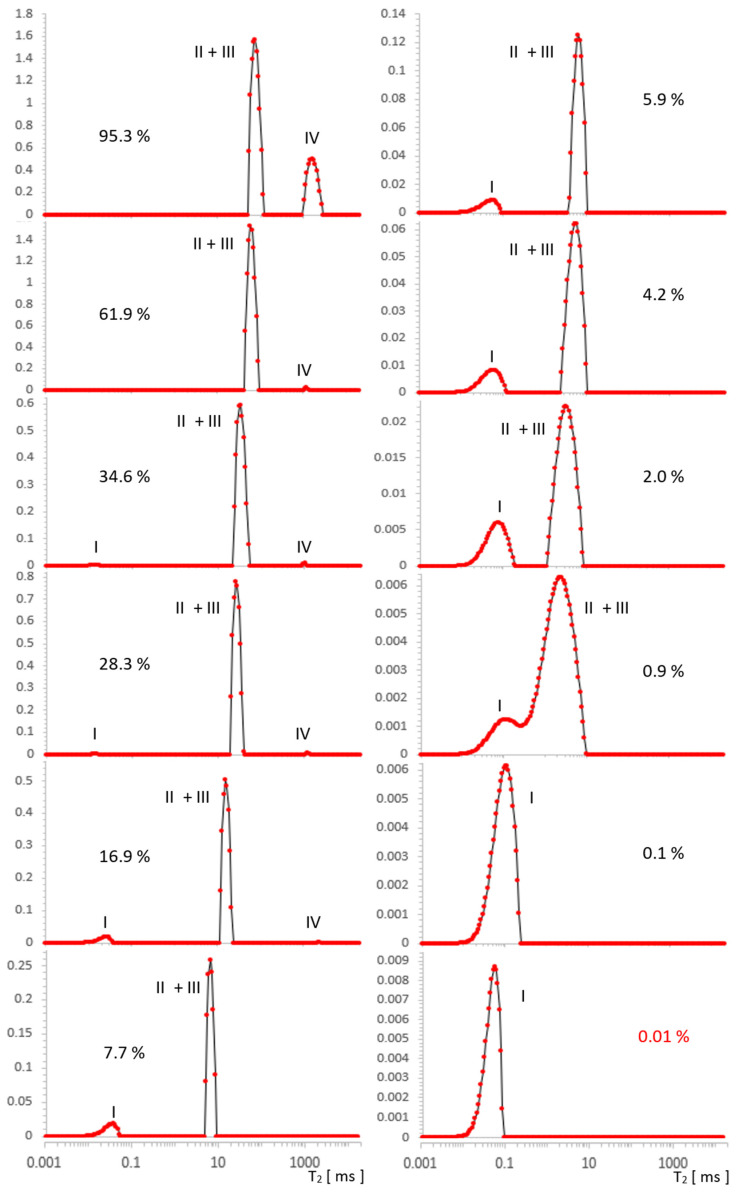
Evolution of the T_2_ spectrum for SBA-15, obtained in [a.u.] using ILT transform, along a series of water saturations. I—OH groups, II—intra pore water, III—inter pore water, IV—free water. The vertical axes represent the values of the ILT transform in [a.u.] units.

**Figure 5:**
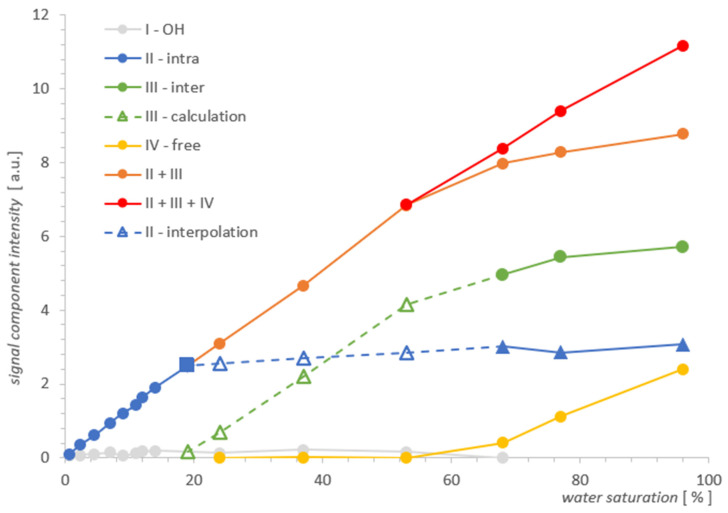
Individual contributions of water in MCM-41 as a function of total water saturation. The square point denotes the water saturation at which the inter water III vanishes, as seen from the comparison of the two spectra for MCM-41 and SBA-15 where both line widths are comparable.

**Figure 6:**
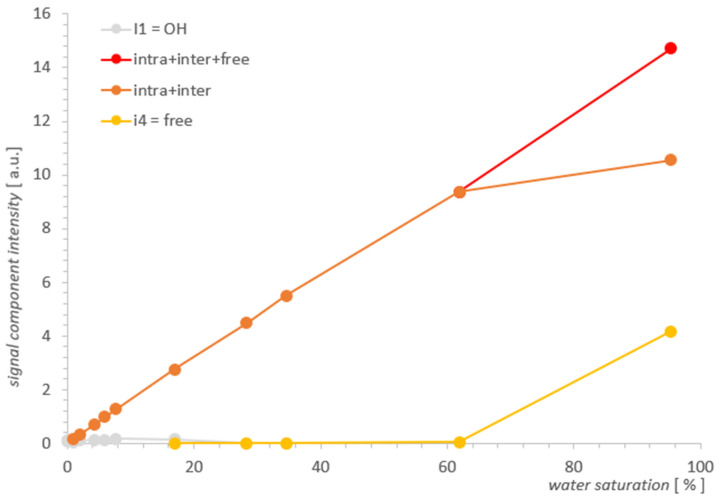
Individual contributions of water in SBA-15 as a function of water saturation.

Spectra T_2_ obtained with L-Curve regularization are shown in Figure 3 for MCM-41 and in Figure 4 for SBA-15. Intensities for different locations found from spectra along a series of water concentrations are presented in Table 1 and Table 2, and then visualized in Figure 5 and Figure 6 for MCM-41 and SBA-15, respectively. By intensity, we understand here as the sum of values in the range containing the maximum of the line (a value with some calculation error in the case of broad, overlapping lines). Respective line positions are summarized in Table A1 and Table A2 in Appendix A for both samples.

### 2.1. Results for MCM-41

In Figure 3, four types of lines can be seen, the intensities of which change as a function of the decreasing water concentration traced in the range 96–0.72%. For the initial maximum concentration of 96%, we distinguished the following contributions in the spectrum: IV—free water around 2000 ms, III—inter water at ~100 ms, II—intra pore water at ~20 ms, and at around 0.06 ms, we had OH groups, which were better visible for lower concentrations.

With drying, the contribution from free water IV noticeably and quickly decreased, and from water inter III, it was relatively slower. It can be expected that the line from water intra II in the pores will begin to disappear when the outer layers are completely removed (i.e., at the latest). Free water IV disappeared around 24% and the line from water inter III completely overlapped that of the water intra II line in pores at a concentration of 53%. This conglomerate of lines II + III, however, loses its intensity successively further, as the water concentration decreases, and it manifests itself essentially by narrowing its width in the T_2_ dimension.

Parallel to the effect of intensity decrease, there is a visible drift of the center of gravity of the complex line II + III toward the shorter T_2_. This drift does not correspond to the actual translation and in this sense is apparent as it can be seen that the left border of the line remained stationary in a wide range of concentrations, which suggests that the component with a shorter T_2_ in the observed sum remains constant in this range or changes little. Therefore, the only cause of the apparent maximum drift seems to be the change in component intensity with a longer T_2_. Altogether, this corresponds to common-sense intuition that water is removed from the sample in a specific order, starting with the geometrically outermost (and perhaps less bound) layers, which could be, for example, a water inter III layer or free water IV as a marker on the surface. On the basis of further analysis, we will argue that this intuition, while essentially correct, is not entirely accurate in this case.

**Figure A1:**
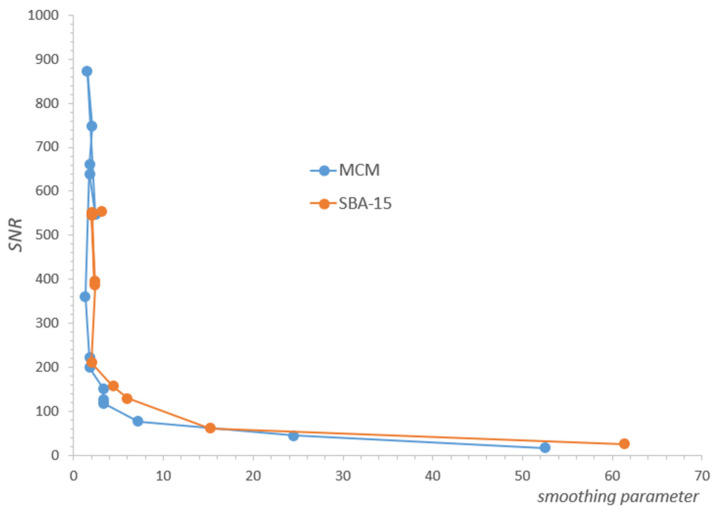
The regularization parameter for ILT transform increases as SNR (Signal-To-Noise-Ratio) decreases.

Due to the overall decrease in water concentration, individual signals in the spectral series also decrease, correspondingly reducing the SNR. The consequences of this fact can be observed independently in the form of increased values of the regularization parameter with a decrease in SNR, found in our analysis for each of the spectra separately using the L-Curve methodology (Figure A1, Appendix A). Such behavior is expected and consistent with ILT properties, and its observation allowed us to control the consistency of the analysis. The decrease in SNR ultimately results in substantial line broadening, an effect that is fundamental to the ILT and is opposite to that caused by water removal. It is noticeable in our spectra, especially at lower concentrations from the value of about 7%, but its direct influence was only a broadening of the spectrum, without affecting the intensity of its individual components, as presented in Table 1 or Table 2.

In the immediate vicinity of the value of 0.06 ms, there was a signal from strongly bound silanol OH groups, which is a separate problem that has already been studied using 2D relaxometry elsewhere [2,24]. It is also the area of possible ILT artifacts due to the time *t_E_* used in the measurements and, due to this value itself, limits the effectiveness of the analysis in this area. As can be seen from Table 1 and Table 2, the intensity of OH I with a change in water concentration slightly oscillated around small values for no apparent reason, which we attributed to the instability of ILT in this area superimposed on a constant and small value of the real signal. This behavior is systematic over the entire range of water concentrations, with the exception of the first two spectra for the strongest signals at the highest concentration, which dominate the other contributions’ intensities for longer T_2_ times. The signal mentioned does not have any influence on the results of this work, nor is it directly related to its topic, therefore we only note here and hereafter its presence, origin, and behavior in the spectra. The spectrum even extends down to 0.02 ms, which is the result of signal extrapolation using ILT in the sense of fitting procedure.

**Figure 7:**
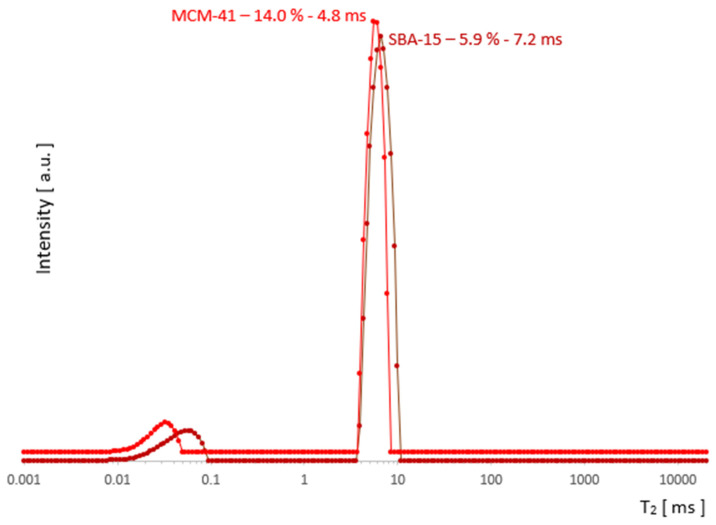
The saturation range for ‘water only in the pores’ (after removal water inter III) begins with the saturation at which the lines for MCM-41 and SBA-15 are similar in width.

The change in the width of a complex line II + III in the ILT spectrum is one of the manifestations of the change in its intensity and this fact, combined with the basic knowledge for both samples, was used to plot the evolution of the components as a function of hydration in Figure 5. It follows from the characteristics provided that the pore diameters, although different, are of the same order in both samples. However, they essentially differed in the size distribution of the particles themselves, which are clusters of nanoparticles. The size distribution of these clusters for MCM-41 varied from very small 0.1 µm to the order of magnitude larger (1 µm), while for SBA-15, it remained on the same order of magnitude of 1–2 µm (see Figure 1). This created more variations in the water inter III distribution for MCM-41 and led to significantly different spectral line widths compared to SBA-15 in the state of full saturation. Such differences can actually be observed in the T_2_ spectra in Figure 3 and Figure 4. On the other hand, we know that both MCM-41 and SBA-15 have comparable pore sizes and similar pore dispersion, so for water intra II there were similar widths of spectral lines at similar T_2_′s. The above premise makes it easy to find the saturation value at which the complex line II + III is devoid of its water inter III component. This is roughly the first saturation toward its decreasing values at which the lines’ widths for both samples are comparable at similar T_2_. The spectra for which this condition holds are shown in Figure 7: at a concentration of 14% for MCM‑41 and 5.9% for SBA-15, these are spectra of almost identical shape and line width. Thus, starting with the saturations mentioned toward their decreasing values, we dealt only with water intra II in the pores in both samples, respectively.

In light of the above, we know that the water inter III vanishing point for MCM‑41 is somewhere between a saturation of 14% and 24%, and we can find the respective signal value for water intra II by interpolation, marked by the square in Figure 5. It is worth noting that the signal at this point does not differ much from its value when the sample is fully saturated, and also when the lines are still split at 77%, we can conclude that the water intra II leaves the MCM-41 sample very slowly with a change in saturation, and the process accelerates dramatically only below 19%. Summarizing the above, the slowly-changing linear interpolation in the area of overlapping spectral lines II and III seems to be a sufficiently accurate approximation for water intra II, as illustrated by the blue dashed line in Figure 5. In the next step, from the simple balance, one can also obtain a plot of the contribution of water inter III in the remaining hydration range (marked with a green dashed line in Figure 5), using the summarized II + III contribution intensity obtained previously from the spectra.

### 2.2. Results for SBA-15

In Figure 4, we can see three types of lines, the intensity of which changes as a function of decreasing water concentration in the range 95.3–0.1% (presented ~0.01% is on the boundary of our accuracy). In the spectrum for the initial maximum concentration of 95.3%, overlapping contributions intra II and inter III can be suspected and are similar to MCM-41 free water IV of about 2000 ms. For MCM-41, the contribution of OH I groups with an artifact superimposed near the beginning of the T_2_ timescale occurs at lower concentrations in SBA-15, which is also attributed to the hydration signal dominating these contributions.

The free water IV contribution decreased significantly around 16.9%, and over the entire saturation range, it seemed to decrease slightly faster with a saturation decrease than in MCM-41.

Following the analogy of MCM-41, here it is also logical to expect intra II and inter III contributions to be present, although starting with maximum hydration, the data seem to contradict this: as an equivalent, we see a well-defined single line across the entire range of saturation. However, this lack of structure turns out to be apparent: for 95.3%, the line position at 77 ms in SBA-15 is, we believe, not accidentally close to the average value of T_2_^II+III^ = 55 ms of the split line positions in MCM 41 for the respective water II and water III contributions, therefore, this line is also complex in SBA-15 and includes these contributions. The position of this line in SBA-15 is between position values for lines II and III in MCM 41 and is relatively narrow, which we attributed to the small spread of the possible water inter III positions in this sample (also consistent with the manufacturer’s information on the small particle size spread).

The individual contributions for SBA-15 are summarized in Table 2 and plotted in Figure 6; these are mainly contributions of water inter II and water intra III. Unlike the MCM-41 case, there is no convincing evidence in the data itself that could help separate overlapping spectral lines.

### 2.3. Discussion

Comparing the results visualized in Figure 5 and in Figure 6 for both samples, the obvious conclusion is that the analytical possibilities extended at the starting point had some effect for MCM-41, but for SBA-15, it proved to be insufficient. The information from the SBA-15 measurements and analysis played an important complementary role in the search for the water inter III elimination point in MCM-41, which was the foundation for determining the remaining contributions as the function of saturation and in the form presented in Figure 5 for MCM-41. However, drawing useful conclusions from the standalone T_2_ spectra for SBA-15 proved to be unfeasible and the resolution improvement too small. As the comparison of SBA-15 and MCM-41 cases suggests, the successful application of the described method to other cases seems to depend on the relative development of the closed surfaces formed by the outer surfaces of adjacent pore aggregates relative to the pore surface.

Dependencies of individual contributions of water on water saturation for MCM-41 in Figure 5 illustrate, as it seems, convincingly, the actual process of water removal from the sample, obtained using T_2_ NMR relaxometry. As the results show, this process takes place simultaneously and not sequentially as one might think, and as we assumed in our previous analysis and measurements [2]. However, the statement made there as an assumption, we get in this approach as a result. The water inter III and free water IV leave the sample simultaneously in the entire range of their presence, having different slopes with respect to the saturation axis in Figure 5. It seems that such a parallel transfer also applies to water from inside the pores intra II, although it happens much slower with a change in saturation and as shown in Figure 5, it accelerates rapidly for the water intra II contribution only at 19%, when the remaining contributions are completely removed. The shape of the curves of the whole process suggests approximately an exponential dependence and a box-like transfer model, in which the movement of water to successive compartments occurs continuously in the entire saturation domain. However, the verification of such a model would require more cases of resolved lines than were available in the prepared set of saturations for MCM-41.

## 3. Conclusions

The main motivation here was to explore the feasibility of analyzing water contributions in a single spectrum and to see if this would add new information from the point of view of a similar experiment performed previously [2]. The premise was the enormous progress that has been made in recent years in the solution of NNLS systems for Laplace analysis and the possibility of the independent implementation of appropriate algorithms. As the example for MCM-41 shows, T_2_ analysis of the contributions in a single spectrum may in principle be feasible and lead to useful results. Rather rough approximations have been used in MCM-41 when analyzing partially overlapping lines, so there is room for further improvement here. In this way, we obtained a picture of the process in Figure 5, which seems to describe the distributions of individual contributions of water in MCM-41 as a function of changes in the total water saturation. The SBA-15 analysis showed the classic and fundamental problems with resolution related to the inherent need for ILT spectra regularization, and this is the case where high resolution methods should be used.

The aforementioned MAS methodology is and will probably remain the gold standard in this type of research due to the excellent differentiation of signals as a function of the chemical environment and both the phenomenal sensitivity and stable and unambiguous results of the analytical procedures (Fourier analysis).

## Figures and Tables

**Table 1:** Contributions to the signal from different water locations in MCM-41 obtained from the respective integrals of the ILT spectra, expressed in [a.u.] for: I—OH groups, II—intra pore water, III—inter pore water, IV—free water.

%	I [a.u.]	II [a.u.]	III [a.u.]	IV [a.u.]
96.0	0	3.0638	5.7096	2.3942
77.0	0	2.8497	5.4366	1.1180
68.0	0.0085	3.0152	4.9602	0.4101
53.0	0.1658	6.8461		0.0108
37.0	0.2163	4.6720		0.0161
24.0	0.1417	3.0997		0.0062
14.0	0.1891	1.9050	-	-
12.0	0.1749	1.6437	-	-
11.0	0.1134	1.4223	-	-
8.9	0.0666	1.2053	-	-
7.0	0.1423	0.9463	-	-
4.5	0.1101	0.6165	-	-
2.3	0.0746	0.3472	-	-
0.7	0.0562	0.1077	-	-

**Table 2:** Contributions to the signal from different water locations in SBA-15 obtained from the respective integrals of the ILT spectra, expressed in [a.u.] for: I—OH groups, II + III—intra- and inter- pore water, IV—free water.

%	I [a.u.]	II + III [a.u.]	IV [a.u.]
95.3	-	10.5503	4.1646
61.9	-	9.3590	0.0429
34.6	0.0057	4.0409	0.0163
28.3	0.0028	4.4826	0.0124
16.9	0.1509	2.7507	0.0027
7.7	0.1768	1.2683	-
5.9	0.1116	0.9836	-
4.2	0.1268	0.7019	-
2.0	0.1003	0.3405	-
0.9	0.0270	0.1540	-
0.1	0.1012		-
~0.0	0.0967		-

## Data Availability

The data presented in this study are available on request from the corresponding author.

## Appendix A

**Table A1:** Line position for MCM-41 in series of water saturations. I—OH groups, II—intra pore water, III—inter pore water, IV—free water.

%	T_2_^I^ [ms]	T_2_^II^ [ms]	T_2_^III^ [ms]	T_2_^IV^ [ms]
96.0	-	16.6275	95.6983	1931.4715
77.0	-	15.9511	90.8495	940.6998
68.0	0.0131	15.6531	96.1959	1595.4480
53.0	0.0212	37.7980		643.4950
37.0	0.0215	16.9998		262.8572
24.0	0.0230	3.0997		305.1463
14.0	0.0289	5.7398	-	-
12.0	0.0259	5.0470	-	-
11.0	0.0345	4.0882	-	-
8.9	0.0427	3.6844	-	-
7.0	0.0515	3.2950	-	-
4.5	0.0809	2.6328	-	-
2.3	0.0840	1.5408	-	-
0.7	0.1091	0.9608	-	-

**Table A2:** Line position for SBA-15 in series of water saturations. I—OH groups, II—intra pore water, III—inter pore water, IV—free water.

%	T_2_^I^ [ms]	T_2_^II+III^ [ms]	T_2_^IV^ [ms]
95.3	-	77.3073	1672.1316
61.9	-	63.1377	1215.8725
34.6	0.0133	33.2756	1008.1585
28.3	0.0121	26.6767	1240.6753
16.9	0.0231	15.8747	2322.2875
7.7	0.0320	7.2114	-
5.9	0.0482	6.6127	-
4.2	0.0554	5.5516	-
2.0	0.0755	3.6012	-
0.9	0.1154	2.5859	-
0.1	0.0984		-
~0.0	0.0500		-
